# Inspiratory muscle training as part of multimodal prehabilitation improves maximal inspiratory pressure in elderly surgical patients: an exploratory secondary analysis of a randomised controlled trial

**DOI:** 10.1186/s12877-026-07944-3

**Published:** 2026-07-07

**Authors:** Simone Wen-Shi Dueblin, Patrick Y. Wuethrich, Dominique Engel, Prisca Eser, Christian M. Beilstein, Matthias Wilhelm, Thomas Vetsch

**Affiliations:** 1 Department of Anaesthesiology and Pain Medicine, Inselspital, Bern University Hospital, University of Bern, Bern, Switzerland; 2 Centre for Rehabilitation & Sports Medicine, Inselspital, Bern University Hospital, University of Bern, Bern, Switzerland

**Keywords:** Prehabilitation, Inspiratory muscle training, Maximal inspiratory pressure, Elderly, Telemedicine

## Abstract

**Background:**

Older adults awaiting major surgery often present with reduced physical fitness and respiratory muscle weakness, which are associated with increased postoperative complications. Prehabilitation may improve functional capacity, but evidence for its effect on inspiratory muscle strength in high-risk older adults remains limited. The main objective was to assess the change in maximal inspiratory pressure (MIP) in the preoperative period in high-risk patients receiving home-based tele-supervised prehabilitation compared to standard care.

**Methods:**

This exploratory analysis included a subset of secondary outcome data from a prospective, two-arm, parallel-group randomised controlled trial conducted at a single university hospital in Switzerland. Patients aged ≥ 65 years awaiting elective cardiac or major non-cardiac surgery with a proven fitness deficit measured by cardiopulmonary exercise testing (CPET) were included. Participants randomized in the intervention arm received a multimodal, home-based tele-supervised prehabilitation programme over 2–4 weeks addressing deficits in physical fitness, nutrition, and anaemia, while controls received standard preoperative care.

**Results:**

Eighty eight participants were analysed (intervention *n* = 48; control *n* = 40). Patients in the intervention group showed improvement in MIP after the training period, whereas the control group demonstrated a decline (median change + 10.5 [0.00; 16.2] vs. – 3.00 [-8.00; 1.50] cmH₂O; *p* < 0.001). After adjustment for baseline MIP, the estimated between-group difference was 13.39 cmH₂O (95%CI 8.12–18.75; *p* < 0.001).

**Conclusions:**

Home-based tele-supervised IMT, delivered as part of a multimodal prehabilitation programme, improved maximal inspiratory pressure over the preoperative period in elderly high-risk surgical patients. The observed MIP decline in the control group suggests that the preoperative waiting period itself may carry a risk of functional deterioration in this population. Whether MIP improvements translate into reduced postoperative morbidity will be addressed by the primary outcome of the ongoing PREHABIL trial.

**Trial registration:**

ClinicalTrials.gov Identifier NCT04461301 (First Submitted: 02.07.2020, First Posted: 08.07.2020).

**Supplementary Information:**

The online version contains supplementary material available at https://doi.org/10.1186/s12877-026-07944-3.

## Introduction

Physical fitness is a key determinant of overall health. Impairment in physical fitness is associated with a greater risk of chronic disease and elevated all-cause mortality [1, 2]. It also accelerates the loss of muscle mass and, muscle strength - both core components of frailty [3]. Notably, reduced physical fitness affects three of the four domains assessed by the Fried Frailty Scale, underscoring its central role in the development and progression of frailty [4].

Frailty has gained growing attention in both clinical and research settings in recent years. It is defined as a multifactorial clinical syndrome characterized by diminished strength, endurance, and physiological reserve, leading to increased vulnerability to dependence and mortality [5]. Frailty is a well-established risk factor for poor postoperative outcomes [6]. Postoperative complications often require additional interventions, which not only impact hospital resources [7], but also negatively affect patients’ overall well-being and quality of life [8].

Frail patients typically present with low physical activity (PA) levels, reduced muscle strength and diminished inspiratory muscle strength. The latter is often reflected in a low maximal inspiratory pressure (MIP) [9, 10]. Studies have shown that low MIP can contribute to poor postoperative outcomes, particularly pulmonary outcomes [11]. Conversely, improving MIP may enhance functional capacity [12, 13].

Prehabilitation is a multimodal intervention concept, designed to improve patient’s general health before surgery, aiming to enhance resilience towards the surgical burden [14–16]. Most previous prehabilitation studies have implemented PA or inspiratory muscle training (IMT) intervention in a supervised in-hospital setting [17, 18]. However, the patients most likely to benefit are often old and frail [19, 20], and consequently frequently face functional, psychosocial, or socioeconomic barriers that make additional hospital visits challenging [21, 22]. Few studies have assessed the effectiveness or feasibility of preoperative multimodal exercise training delivered in a home setting [14, 23]. 

Therefore, the aim of this exploratory analysis was to investigate whether a home-based, tele-supervised multimodal intervention - including IMT - could improve MIP preoperatively in elderly patients with documented exercise-deficit scheduled for major elective surgery.

## Methods

This study is an exploratory sub-analysis of the PREHABIL trial and based on preliminary secondary outcome data [24]. The PREHABIL trial was conducted in accordance with the Good Clinical Practice recommendations of the Declaration of Helsinki, approved by the Ethics Committee of the Canton of Berne, Switzerland (Kantonale Ethikkommission Bern, KEKBE 2020 − 01690), and registered at ClinicalTrials.gov (NCT04461301, First Submitted: 02.07.2020, First Posted: 08.07.2020). Written informed consent was obtained from all participants before enrolment. This was a multidisciplinary, prospective, 2-arm, parallel group, randomized, controlled superiority trial with an allocation ratio of 1:1 and a blinded outcome assessment. Due to the nature of the exercise intervention, blinding of participants and the study team members delivering the prehabilitation programme was not feasible. The statistician performing the primary analysis was blinded to group allocation.

The published protocol provides detailed descriptions of the design, recruitments, and data collection procedures [24]. Adverse events were defined as any untoward medical occurrence during the prehabilitation period and were ascertained at the preoperative study visit by study team members.

The screening visit comprised a cardiopulmonary exercise test (CPET), body composition assessment, and basic bloodwork. After completion of the screening visit, patients who fulfilled the inclusion criteria (> 65 years, planned major visceral, urologic, cardiac, vascular, orthopaedic or thoracic surgery, ≥ ASA III physical score, proven deficit in functional capacity) were assigned a study ID and then randomized using a minimization algorithm either to the intervention arm (prehabilitation) or the control arm (standard of care). The minimization algorithm assures a balanced randomization of included surgical disciplines (i.e., equal distribution of patients undergoing same-day surgery or getting admitted the day before surgery). The intervention arm includes individualized nutritional recommendations, tailored physical exercise, correction of anemia (patient blood management) and smoking cessation counselling. The exercise program consisted of strength, endurance (primarily walking) and respiratory exercises tailored to each patient’s capabilities. The control arm consisted of standard care as pre-determined by each surgical department [24].

While some patients were not able to conduct aerobic exercise due to severe musculoskeletal conditions, all patients were prescribed IMT using a mechanical IMT device (POWERbreathe Medic Plus, healthmg, Hirzel, Switzerland). Patients were instructed to train twice daily for 30 inspirations each time starting at an initial training load of 40% of the initially measured MIP measured by MicroRPM Respiratory Pressure Meter (MD Spiro, Lewiston, Maine, USA). To support adherence to the program, a study team member contacted participants weekly by telephone calls to adjust the PA program and address any questions or concerns. PA recommendations were given to the patients according to the Swiss Federal Office of Health in either French or German, advising either ≥ 150 min/week of moderate–intensity aerobic activity or ≥ 75 min/week of vigorous-intensity activity. If the patient subjectively considered the program as low effort (BORG scale < 13), the intensity was adjusted by increasing the resistance of the IMT device or by increasing training loads of the endurance and/or strength training. No in-person supervision was provided.

### Baseline and preoperative assessment

Patients were assessed twice during the preoperative period. Once at inclusion (baseline) and again after 2–4 weeks on the day before surgery (preoperative). At baseline, socio-demographic data were also collected, including pre-existing comorbidities and medications. Additionally, blood was sampled at baseline for detecting any nutritional deficits. Grip strength (Hydraulic Hand Dynamometer, Jamar Health Products Inc., Greendale, USA) and MIP (MicroRPM Respiratory Pressure Meter, MD Spiro, Lewiston, Maine, USA) were measured in all patients during the baseline visit and the preoperative assessment. PA was assessed by wrist-worn accelerometry (Axivity AX-3, Axivity Ltd., Newcastle upon Tyne, UK) during the whole preoperative period from which parameters such as steps per day and time of moderate-to-vigorous PA were quantified.

### Statistical methods

All analyses were performed with R [25]. Descriptive statistics were used to summarize the baseline patient characterized. To assess whether the distribution of the changes in MIP was approximately normal, we conducted the Shapiro-Wilk test for normality in each randomization group. Non-normality was indicated in the control group (*p* = 0.03) but not in the intervention group (*p* = 0.76). Therefore, we assessed between-group difference of changes in MIP by Wilcoxon two sample test. Corresponding 95% confidence interval and effect size (r) were calculated using the rstatix package using a bootstrapping approach [26]. Baseline characterized were also tested between groups by t-tests, Wilcoxon two sample tests or Chi square tests, as appropriate.

### Sample size

The parent PREHABIL trial is powered to detect a clinically relevant difference in the Comprehensive Complications Index (CCI) as its primary endpoint (target *n* = 466). The present sub-analysis was not independently powered.

## Results

**Fig. 1:**
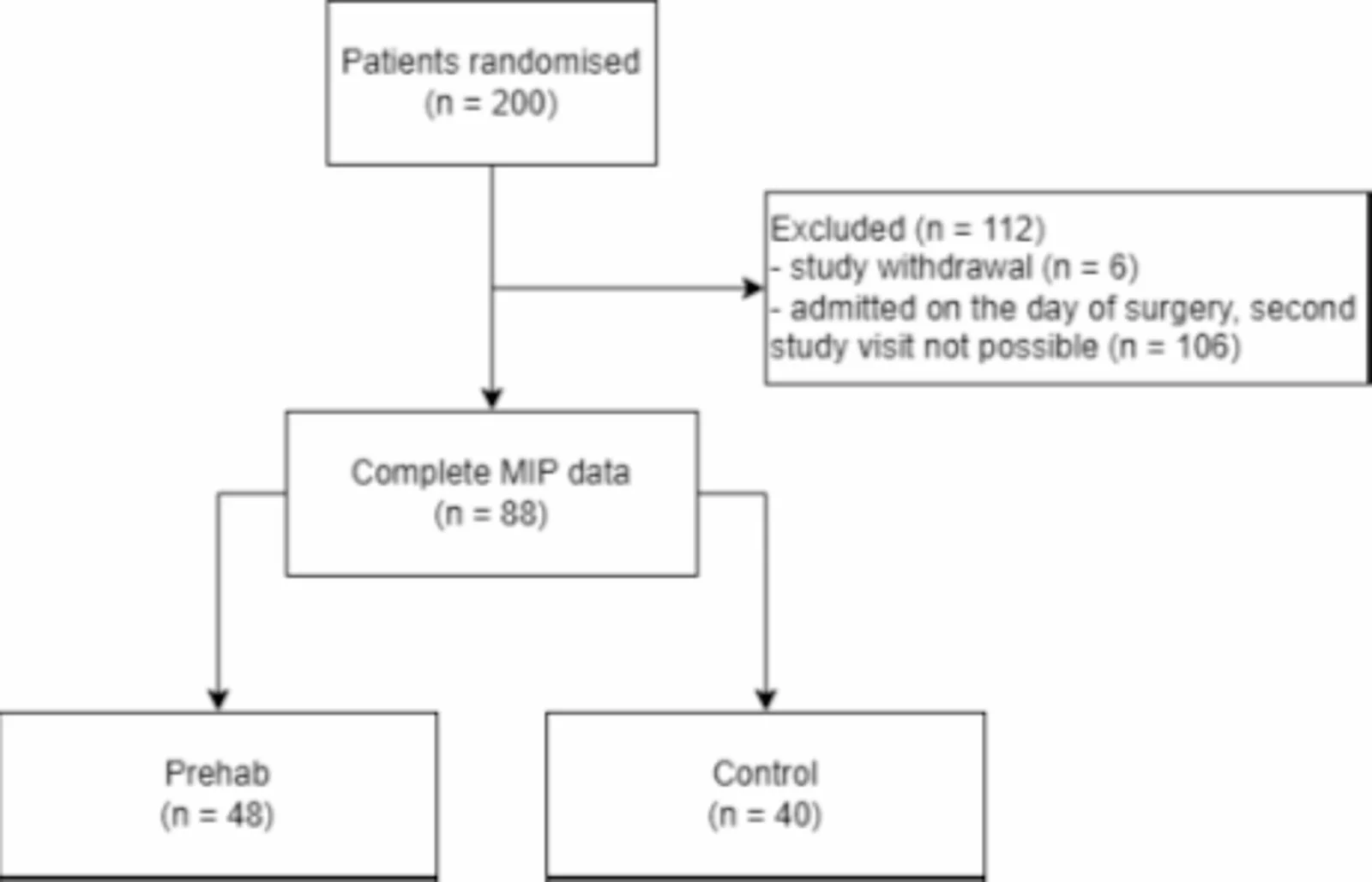
Patient flow

Between 02/2022 and 12/2024, 200 patients (of the planned 466 for the ongoing PREHABIL study) were recruited in the main trial, of these 6 withdrew from the study and 106 had no pre-operative MIP measurement because they were admitted to hospital the same day of surgery and the preoperative study visit was not feasible. Thus, 88 had a complete dataset with baseline and preoperative MIP assessments. Of these, 40 were randomized to the control group and 48 to the intervention group (Fig. 1).

Baseline characteristics of the patients included in the analysis of MIP data are summarized in (Table 1). There were no significant differences between the groups.

**Table 1:** Baseline characteristics of control and prehab group

	prehab	control	*p* value
	*N* = 48	*N* = 40	
Patient characteristics			
Age	75.6 (5.75)	75.8 (6.67)	0.871
Sex			0.691
Female	9 (18.8%)	10 (25.0%)	
Male	39 (81.3%)	30 (75.0%)	
BMI [kg/m^2^]	28.6 (5.52)	28.7 (5.91)	0.920
Surgery			0.546
Cardiac	21 (43.8%)	21 (52.5%)	
Non-cardiac	27 (56.2%)	19 (47.5%)	
Type of surgery			0.621
Cardiac	21 (43.8%)	21 (52.5%)	
Orthopedic	4 (8.33%)	5 (12.5%)	
Thoracic	1 (2.08%)	1 (2.50%)	
Urologic	6 (12.5%)	6 (15.0%)	
Vascular	11 (22.9%)	6 (15.0%)	
Visceral	5 (10.4%)	1 (2.50%)	
Days to surgery	33.2 (20.6)	29.9 (14.6)	0.393
Physical function			
NYHA Score	2.25 (0.56)	2.02 (0.58)	0.069
Grip strength [kg]	26.4 (9.07)	28.0 (9.04)	0.427
Fried Frailty Score	1.73 (1.20)	1.25 (1.28)	0.075
Mean steps per day	5630 (2922)	5605 (3582)	0.973
Time of moderate PA [min]	118 (83.6)	113 (69.7)	0.766
MIP			
Baseline MIP	53.4 (21.7)	60.0 (28.0)	0.230
CPET data			
METS	5.25 (1.11)	5.70 (1.70)	0.159
FVC [L]	3.07 (0.82)	3.36 (0.89)	0.121
FEV_1_ [L]	2.23 (0.62)	2.48 (0.69)	0.083
Power peak [watt]	68.8 (28.9)	79.0 (33.6)	0.146
VO_2_@VT_1_ [mL/min/kg]	11.5 (2.57)	11.5 (2.81)	0.921
VO_2_@peak [mL/min/kg]	14.5 (3.18)	15.2 (4.16)	0.404
VE/VCO_2_ slope	39.7 (7.16)	40.7 (6.76)	0.533
Blood sampling			
Hemoglobin [g/L]	128 (19.3)	133 (14.2)	0.130
Glucose [mmol/L]	7.21 (3.38)	6.81 (1.63)	0.496
Potassium [mmol/L]	4.26 (0.46)	4.17 (0.32)	0.336

*Abbreviations*: *BMI *body mass index, *NYHA *New York Heart Association, *PA *physical activity, *MIP *maximal inspiratory pressure, *CPET *cardiopulmonary exercise testing, *METS *metabolic equivalents, *FVC *forced vital capacity, *FEV*_1_ forced expiratory volume in 1 s *VO*_2_@VT1 oxygen uptake at first ventilatory threshold, *VO*_2_@peak  oxygen uptake at peak exercise

**Fig. 2:**
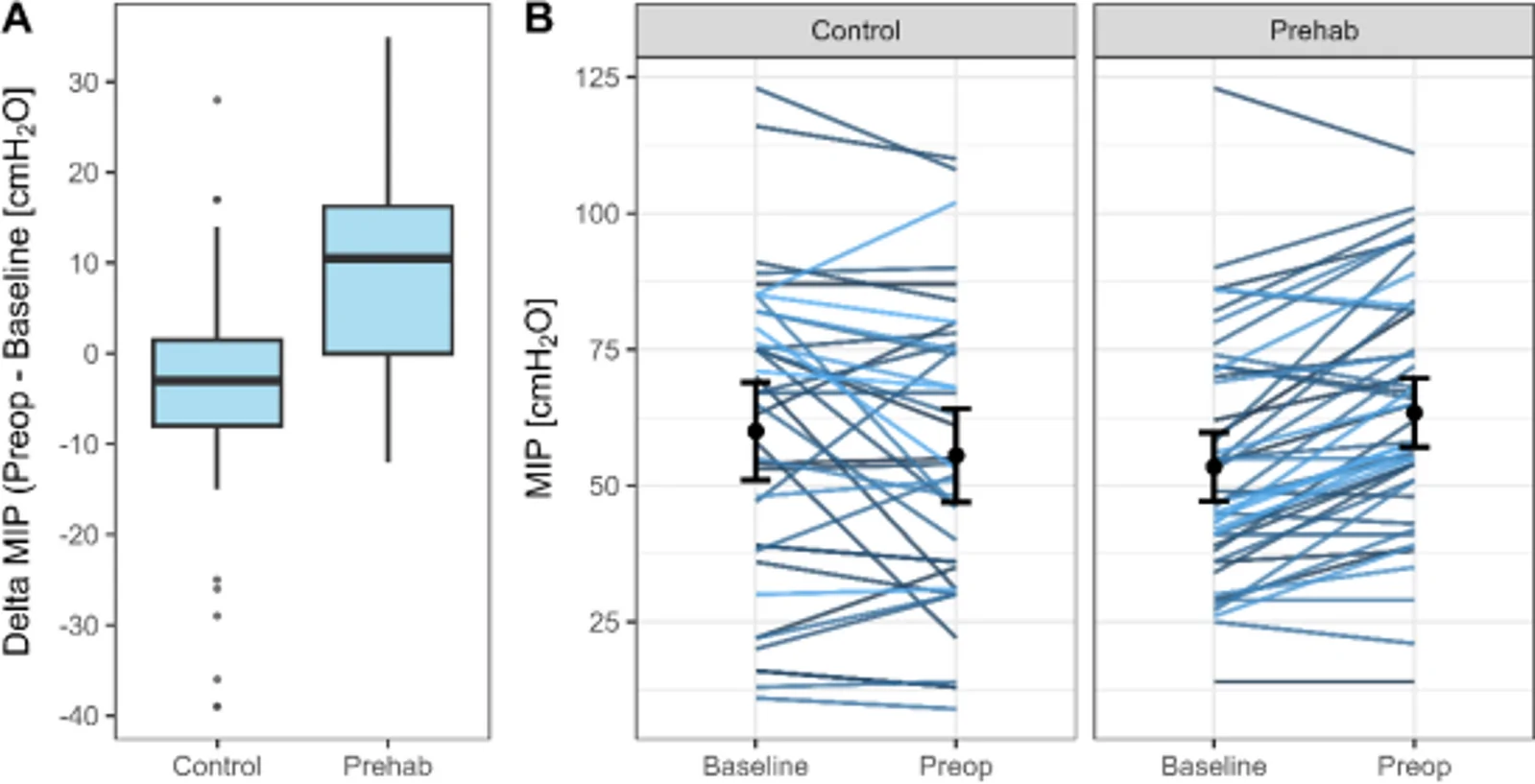
**A** Change in MIP between the prehab and the control group (*p* < 0.001). **B** Individual changes in MIP as well as mean and standard errors at both time points of the prehab and control group

Figure 2 compares the changes in MIP from baseline visit to preoperative visit between the prehab and the control group. Median MIP values declined over time in the control group from 66.0 [38.8; 79.8] cmH_2_O to 53.5 [34.0; 75.2] cmH_2_O (delta MIP control = – 3.00 [− 8.00; 1.50] cmH_2_O), while they improved in the prehab group over time from 48.0 [38.8; 70.2] cmH_2_O to 60.0 [51.0; 76.8] cmH_2_O (delta MIP prehab = 10.5 [0.00; 16.2] cmH_2_O); (Fig. 2A). The difference in median delta MIP of 13 cmH_2_O (95% CI 8–19 cmH_2_O) between groups was statistically significant (*p* < 0.001), with moderate effect size (*r* = 0.49). These findings were further supported by an ANCOVA adjusting for baseline MIP values, which confirmed a significant effect of the prehabilitation intervention (b = 13.39 cmH₂O, 95% CI [8.12; 18.75], *p* < 0.001; Supplementary Table S1). Individual changes of both groups are shown in (Fig. 2B). A Wilcoxon signed-rank test against zero showed that the within-control-group change did not differ significantly from zero (V = 251.5, pseudo-median − 3.50 cmH₂O, 95% CI [− 8.00; 1.00], *p* = 0.085), indicating that the decline in the control group was not robustly established as a within-group finding.

To contextualise the clinical relevance of MIP as a preoperative assessment tool, cross-sectional associations between baseline MIP and measures of physical function and cardiorespiratory fitness were examined. Baseline MIP showed moderate positive correlations with grip strength (*r* = 0.53, 95% CI 0.36–0.66), FVC (*r* = 0.4, 95% CI 0.21–0.57), VE at peak exercise (*r* = 0.5, 95% CI 0.33–0.65), and VO₂ at peak exercise (*r* = 0.36, 95% CI 0.16–0.53; all *p* < 0.001; Supplementary Figure S2).

No serious adverse events related to the prehabilitation were recorded during the preoperative training period.

## Discussion

The novelty of our study was the delivery of IMT implemented in multimodal, home-based tele-supervised prehabilitation program specifically tailored for the elderly patient with a proven fitness deficit. We observed an improvement in median MIP by 13 cmH_2_O in the preoperative period after an averaged training period of 33.2 days with IMT compared to no respiratory training. This between-group difference remained significant after adjustment for baseline MIP values in an ANCOVA, confirming that the observed effect was not attributable to baseline MIP differences between groups. The apparent decline in the control group (median − 3.00 [− 8.00; 1.50] cmH₂O) did not reach statistical significance when tested against zero (*p* = 0.085), and the confidence interval straddles zero, so caution is warranted in interpreting this as a true within-group deterioration. Measurement variability inherent to the MIP testing is a plausible alternative explanation. A familiarisation effect would not account for the observed direction of decline, since there would be an expected shift of scores upward rather than downward. That said, functional deterioration during the preoperative waiting period in frail older adults is biologically plausible and consistent with the broader literature on disuse-related muscle atrophy [27]. Reduced physical activity and preoperative anxiety are contributing factors that cannot be excluded. Crucially, the highly significant between-group difference (*p* < 0.001) does not depend on the control-group decline being a true effect. It reflects the contrast between a group that trained and one that did not, and this contrast remains robust regardless of the interpretation of the within-control trajectory.

The improvement in the intervention group is consistent with findings from comparable trials: Akowuah et al. reported similar improvement of 10.6 cmH_2_O, while Hulzebos et al. saw an improvement of 14.5 cmH_2_O in in-person supervised or semi-supervised settings [17, 28].

Whether our MIP improvement of 13 cmH_2_O is clinically relevant remains open for debate. The minimal clinical important difference (MCID) of MIP changes has not been reported in a surgical population with an exercise deficit. Beaumont et al. estimated an MCID between 7.9 and 13.5 cmH_2_O in a COPD population after having completed a respiratory rehabilitation of 4 weeks [29] with them making the suggestion to make the cut-off for the MCID on the higher range at 13.5 cmH_2_O. Our observed improvement of 13 cmH₂O falls within this range, suggesting potential clinical relevance, although caution is warranted when extrapolating MCID estimates across populations.

Beyond the question of clinical relevance, the implications of MIP improvement for postoperative outcomes are of particular interest. Previous studies have found an improvement of MIP to be correlated with a reduction in pulmonary complications after major surgery, as well as length of hospital stay [28, 30]. Whether the MIP gains observed in our population translate into tangible reductions in postoperative morbidity will be addressed by the primary outcome of the ongoing PREHABIL trial, which uses the Comprehensive Complications Index (CCI) as its primary endpoint.

Irrespective of its responsiveness to training, MIP has important characterized that make it attractive as a clinical assessment tool. It is measured non-invasively using a handheld device, requires minimal patient effort, and can be completed within minutes. In this regard, MIP parallels grip strength as a simple bedside surrogate of overall physical capacity, with the added value of specifically reflecting pulmonary function and respiratory muscle reserve. This dual role, as both a training target and a clinical marker, supports its broader integration into preoperative risk assessment.

This perspective is supported by the moderate correlations observed at baseline between MIP and spirometric parameters (FVC) and cardiorespiratory fitness markers (VO₂ at peak exercise, VE at peak exercise), which are consistent with prior reports in COPD and abdominal surgery populations [31–33].

The moderate correlation between MIP and grip strength aligns with findings by Enright et al. and supports the interpretation that peripheral and respiratory muscle strength reflect a shared underlying mechanism. In this context, MIP may serve as a complementary clinical marker in frailty assessment, reflecting respiratory reserve alongside peripheral muscle strength, a relationship supported by Vidal et al. [34, 35].

Altogether, these findings suggest that IMT is a feasible and potentially effective component of multimodal prehabilitation in elderly surgical patients, and that MIP may warrant greater attention as both a training target and a clinical marker in this population.

### Limitations

Several limitations of this study warrant consideration. First, this analysis is based on a complete-case subset of 88 out of 200 randomised patients, as 106 participants had no preoperative MIP measurement available. The primary reason for this was a logistical one: whether a preoperative study visit was feasible depended on the admission practice of the respective surgical department. Departments admitting patients one day prior to surgery allowed time for a second study visit, whereas departments admitting patients on the day of surgery did not. Although this mechanism operated primarily at the departmental level and affected both study arms equally, the resulting sample may not be fully representative of the overall trial population, and a degree of selection bias cannot be excluded.

Second, as this sub-analysis was not independently powered, the available sample of 88 patients was not formally designed to detect a specific between-group difference in MIP. However, the detection of a statistically significant effect within this relatively small sample suggests an effect of meaningful magnitude, as a larger sample would have been required to detect a smaller, potentially clinically marginal effect.

Third, participants could not be blinded to group allocation due to the nature of the exercise intervention. A performance bias cannot be fully excluded.

Fourth, the intervention arm received a multimodal prehabilitation programme comprising physical exercise, nutritional counselling, anaemia correction, and smoking cessation counselling in addition to IMT. Given this design, it is not possible to attribute the observed MIP improvement specifically to IMT, as improvements in general fitness, nutritional status, or structured weekly contact may have contributed.

Finally, compliance with IMT was assessed subjectively based on participant self-report. While participants consistently reported daily use of the device, which they described as easy to incorporate into their daily routine, no objective data such as electronic logging were available to verify adherence.

## Conclusion

Home-based tele-supervised IMT, delivered as part of a multimodal prehabilitation programme, improved maximal inspiratory pressure over the preoperative period in elderly high-risk surgical patients. The observed decline in the control group suggests that the preoperative waiting period itself may carry a risk of functional deterioration, further underscoring the rationale for active prehabilitation in this population. Baseline MIP showed moderate correlations with grip strength and markers of cardiorespiratory fitness, supporting its potential role as a complementary clinical marker in frailty assessment. Whether MIP improvements translate into reduced postoperative morbidity will be addressed by the primary outcome of the ongoing PREHABIL trial. 

## Supplementary Information

Supplementary Material 1.

## Data Availability

The datasets used and/or analysed during the current study are available from the corresponding author on reasonable request.

## Declarations

The protocol was published in BMJ Open [24].
