# Supplementary material for: Inspiratory muscle training as part of multimodal prehabilitation improves maximal inspiratory pressure in elderly surgical patients: an exploratory secondary analysis of a randomised controlled trial
Source: BMC Geriatr. 2026 Jul 7;26:1237. doi: 10.1186/s12877-026-07944-3 (PMC13629078; doi:10.1186/s12877-026-07944-3)
Supplement: Supplementary file 1 [file 12877_2026_7944_MOESM1_ESM.docx]

Supplementary file

**Inspiratory Muscle Training as Part of Multimodal Prehabilitation Improves Maximal Inspiratory Pressure in Elderly Surgical Patients: An Exploratory Secondary Analysis of a Randomised Controlled Trial**

Simone Wen-Shi Dueblin ^1^, Patrick Y. Wuethrich ^1^, Dominique Engel ^1^, Prisca Eser ^2^, Christian M. Beilstein ^1^, Matthias Wilhelm ^2^, Thomas Vetsch ^1, 2^

^1^ Department of Anaesthesiology and Pain Medicine, Inselspital, Bern University Hospital, University of Bern, Switzerland

^2^ Centre for Rehabilitation & Sports Medicine, Inselspital, Bern University Hospital, University of Bern, Switzerland

| Parameter | Coefficient | 95% CI | p |
| --- | --- | --- | --- |
| (Intercept) | 4.86 | [-1.18, 10.94] | 0.122 |
| baseline MIP | 0.85 | [ 0.76, 0.93] | < .001 |
| prehab | 13.39 | [ 8.12, 18.75] | < .001 |

***Supplementary Table S1.*** *After adjusting for baseline MIP values using an ANCOVA, there was a significant effect of the prehab intervention (b = 13.39, 95% CI [8.12, 18.75], p < .001). Patients in the prehab group achieved significantly higher MIP scores compared to the control group, with an average adjusted advantage of 13.39 cmH2O.*

***Supplementary Figure S2.*** *Scatterplots of baseline MIP versus baseline grip strength (upper left), FVC (upper right), ventilation at peak exercise (lower left) and VO_2_ at peak exercise (lower right). Univariate regression lines with 95% confidence intervals are shown in blue.*
